# To seek or not to seek: decision-making in perceived social support among bullied adolescents

**DOI:** 10.1186/s40359-026-04474-w

**Published:** 2026-04-01

**Authors:** Felicia Huang, Ruiqi Lu, Harold Chui

**Affiliations:** 1 Jockey Club School of Public Health and Primary Care, Chinese University of Hong Kong, Hong Kong, China; 2 Department of Educational Psychology, Chinese University of Hong Kong, Hong Kong, China

**Keywords:** School bullying, Social support, Decision-making, Adolescent

## Abstract

**Background:**

Higher perceived social support is associated with reduced psychological and behavioral problems among bullied adolescents; however, many decide not to seek support. Understanding how bullied adolescents make the decision to seek social support is crucial for encouraging support-seeking behaviors. Existing research often examines single factors of perceived social support in isolation or describes decision-making processes not specifically relevant to bullied adolescents. This study adopts a holistic perspective to explore the contributing factors and their interactions, providing a comprehensive account of how bullied adolescents make decisions about seeking social support.

**Methods:**

Ten bullied adolescents (five girls, five boys; *M*age = 14.00, *SD* = 0.47) from China were interviewed. Interpretative phenomenological analysis (IPA) was employed to investigate their lived experiences of seeking or not seeking social support.

**Results:**

IPA revealed four factors to seeking social support among bullied adolescents: (1) Prefer to use personal coping strategies, (2) determine whether the benefits of support-seeking outweigh the risks, (3) estimate if at least one of the three thresholds of support-seeking is reached, and (4) evaluate the effectiveness of support-seeking based on responses received and their effects. Notably, the interactions among these factors significantly shaped their decision-making process. From these insights, we developed a conceptual model that illustrates how bullied adolescents navigate the decision to seek social support.

**Conclusions:**

The findings emphasize that the decision-making process regarding social support among bullied adolescents is multifaceted, shaped by a variety of interconnected factors. This integrated model provides a comprehensive framework for understanding and promoting support-seeking behaviors in this vulnerable population.

**Supplementary Information:**

The online version contains supplementary material available at 10.1186/s40359-026-04474-w.

Research consistently demonstrates that higher perceived social support is linked to fewer subsequent school bullying incidents and reduced psychological and behavioral problems among bullied students [1–3]. Nevertheless, not all bullied adolescents seek social support, potentially exposing them to heightened risks of ongoing bullying and psychological issues [1, 4]. Understanding how these adolescents decide to seek social support can help us encourage support-seeking behaviors and offer timely and efficient support. Thus far, empirical research has mainly looked at discrete factors that influence support-seeking [5], which constrains our understanding of the interaction among factors and a fuller picture of bullied adolescents’ decision-making process [6]. Various theoretical models of support-seeking have been proposed, but few specifically address the unique challenges faced by bullied adolescents. Therefore, this study explored the factors that bullied adolescents consider when deciding on their coping strategies, with particular attention paid to the interactions between these factors as they move toward or away from seeking social support.

## Unwillingness to seek social support among bullied adolescents

This study emphasizes the subjective perception of social support. Therefore, we adopt the following definition of social support: “an individual’s perceptions of general support or specific supportive behaviors from people in their social network, which enhances their functioning or may buffer them from adverse outcomes” [7 p. 215]. While anti-bullying interventions consistently encourage bullied adolescents to seek support, many of them decide not to do so [8]. A wave of studies has been undertaken to investigate this phenomenon and identify several factors. For example, bullied adolescents are less willing to seek social support, if they believe that their own coping strategies work better [9, 10], that bullying incidents and their impacts are manageable [11, 12], that seeking support has more drawbacks (e.g., stigma) than benefits (e.g., emotional comfort) [3–5], or that their prior attempts at support-seeking are ineffective [10, 13].

While these studies shed light on the factors behind bullied adolescents’ decisions toward seeking support, they fall short of offering a comprehensive understanding of how these adolescents navigate the support-seeking process, as the relationships among these factors remain unclear. Having a better understanding of the interplay between the contributing factors is essential to delineate a thorough framework that describes the decision-making process of bullied adolescents and helps in better leveraging the role of perceived social support in future school bullying interventions.

## Theoretical perspectives on support-seeking behavior

A variety of behavioral models have been developed to explain why individuals seek support [14]. For example, the Theory of Reasoned Action [15] and the Theory of Planned Behavior [16] describe a general process in which attitudes, subjective norms, and perceived behavioral control influence one’s motivation to engage in specific behaviors. Other models focusing specifically on young people’s support-seeking have also been proposed. For example, Barker et al. [17] introduced a framework that distinguishes between individual and structural determinants of support-seeking behavior, underscoring influences at the individual, structural, and program levels. Additionally, Biddle et al.’s [18] Cycle of Avoidance Model and Rickwood et al.’s [19] framework concentrate on mental health support-seeking specifically.

These models offer valuable insights into support-seeking behaviors, but they have notable limitations in the context of bullying. While seeking support may appear to be a straightforward decision to make, those experiencing bullying must navigate several obstacles. Prior models often overlook the unique emotional and social dynamics that bullied adolescents face. A frequently reported challenge is the fear of retaliation from bullies [20, 21]. Adolescents often worry that even if they work up the courage to report the bullying, it will not stop; instead, it could intensify, placing them in even more difficult circumstances [21]. Moreover, social stigma is commonly associated with bullying. This hesitation to seek help particularly prevalent among adolescent boys, who fear that reaching out for help, rather than enduring the situation alone, may be regarded as a sign of weakness and incompetence by their peers [10, 22]. Despite considerable efforts to identify and examine the factors influencing support-seeking behavior among adolescents [10, 21], most existing models discuss general support-seeking process but limit in their abilities to account for the unique emotional and social barriers that bullied adolescents face, such as fear of retaliation and social stigma [6]. This gap highlights the need for more integrative models that explicitly consider the context of bullying when delineating the support-seeking process.

The transactional theory of stress and coping [23] posits that individuals engage in primary and secondary cognitive appraisals when faced with stressful situations. The primary appraisal determines whether a situation is threatening, and the secondary appraisal ascertains whether the situation is manageable given the coping resources available [23]. Notably, these appraisals do not occur in isolation; they interact intricately to shape individuals’ decisions on coping strategies. In the context of school bullying, adolescents appraise whether their circumstances are benign or threatening to their personal well-being (primary appraisal) and whether they can cope using available personal and social resources (secondary appraisal). This process ultimately shapes their coping strategies. Furthermore, primary and secondary appraisals are influenced by both individual factors (e.g., personal values and beliefs) and environmental factors (e.g., peer group culture and school climate). Of note, the transactional theory of stress and coping acknowledges the cognitive appraisals that bullied adolescents engage in while navigating bullying situations and allows for an exploration of how individual and environmental factors shape these appraisals. Inspired by this theory and the existing literature on school bullying and support-seeking behavior, our study takes a holistic perspective to investigate the decision-making process that adolescents undergo when determining whether to seek social support in response to school bullying.

## Method

### Present study

Research demonstrates that perceived social support is associated with reduced school bullying and its adverse impacts, but many bullied adolescents refrained from seeking support [4]. Despite increased research into this phenomenon, most studies have examined the factors to perceived social support in isolation, which prevents us from obtaining a fuller picture of bullied adolescents’ decision-making process because it is unclear how these factors interact [6]. The goal of this study was to take an initial step toward filling this gap by providing a more complete account of how bullied adolescents make decisions toward seeking support. In particular, we used qualitative interviews to delve into the factors that bullied adolescents take into consideration when deciding on their coping strategies, with particular attention paid to the interactions between these factors as adolescents move towards or away from seeking social support. Such knowledge is essential for developing a comprehensive model that illustrates bullied adolescents’ decision-making processes, ultimately enhancing the design of programs to promote support-seeking in these individuals.

### Participants

Ten Chinese students (five girls, five boys; age *M* = 14.00, *SD* = 0.47) from three public middle schools located in urban areas of Gansu Province, a northwestern region of China, were interviewed in this study. To limit potential retrospective recall bias, participants were recruited based on recent or ongoing bullying experiences. At the time of the interview, seven participants reported recent victimization in the current semester, and three reported that their bullying victimization was ongoing. Four of the participants reported experiencing verbal victimization only and the remaining participants reported experiencing multiple types of victimization (e.g., physical, verbal, indirect, and cyber). All but one participant reported experiencing chronic victimization, where the victimization occurred repeatedly over a semester. After being bullied, all participants reported to be emotionally impacted, such as experiencing fear, insecurity, or anxiety. Additionally, some participants reported social impacts (*n* = 5), such as a lack of trust in friends, and behavioral impacts (*n* = 4), such as insomnia.

#### Sources of perceived social support

Nine participants reported to have reached out for social support. All of them reported having sought support from family members, including father and mother (*n* = 5), mother only (*n* = 2), grandmother (*n* = 1), and sibling (*n* = 1). Eight participants stated to ask their close friends for help. Four participants reported to turn to their teachers for support, with two seeking help from teachers directly and two doing so indirectly through their parents or classmates. Only one participant reported to have sought classmate support. Details regarding each participant are provided in Table 1.

**Table 1:** Participants’ demographic, experience of bullying victimization, and sources of social support

Participant	Gender	Grade	Type of Victimization	Frequency of Victimization	Impact of Victimization^a^		Sources of Social Support^b^	
1. Jack	Boy	8	Verbal	Sporadic		E, S		F, T, CF
2. Nina	Girl	8	Physical, verbal	Chronic		E, S, B		F, T, CF
3. Rose	Girl	8	Verbal, indirect, cyber	Chronic		E, S, B		F, CF
4. Maria	Girl	9	Indirect, verbal	Chronic		E, S		F, CF, C
5. Anna	Girl	8	Physical, verbal	Chronic		E		T, CF
6. Ryan	Boy	8	Verbal, cyber	Chronic		E		CF
7. Dawson	Boy	8	Physical, verbal, indirect, cyber	Chronic		E, S, B		F, T, CF
8. Zoey	Girl	8	Verbal	Chronic		E		F, CF
9. Jason	Boy	8	Verbal	Chronic		E		F, T
10. David	Boy	8	Verbal	Chronic		E, B	None Reported	

^a^E = Emotional, S = Social, B = Behavioral; ^b^F = Family, T = Teacher, CF = Close friend, C = Classmate

#### Family background

The ten adolescents came from diverse family backgrounds. At the time of the study, all participants reported living in two-parent households. Regarding parental education, 13 parents attained higher education degrees and seven completed secondary education. In terms of employment status, 17 parents were employed in full-time or part-time positions, while three were unemployed or engaged in informal work.

### Researchers

The three authors of this study have different training and research experience in school bullying and IPA. The first author is a female researcher in developmental psychology with experience in adolescent mental health, school bullying, help-seeking, and perceived social support. This background provided familiarity with the phenomena but also carried the risk of bringing presumptions that could shape the interpretations. To mitigate potential biases, she adopted a “not knowing” and curious stance to approach participants’ narratives [24]. The second author is a female researcher trained in counseling and psychotherapy, with prior experience conducting IPA studies. Before analysis began, the first and second authors held reflexive discussions to review IPA theoretical foundations, clarify its differences from other qualitative approaches, and reflect on how they understood each analysis step. They also acknowledged that, as adults interpreting adolescents’ sensemaking of bullying and support-seeking, they may bring adult perspectives that could shape understanding of peer dynamics, power dynamics, and coping. The third author is a male counseling psychologist and the academic supervisor of the first two authors, with substantial experience in school mental health research. Although his seniority and mentoring role may have influenced aspects of the analytic decisions, his role focused on methodological guidance and auditing rather than direct analysis of data. His involvement helped ensure rigor by questioning preliminary themes, prompting justification of interpretations, and reviewing the manuscript for coherence.

### Measures

#### Questionnaire

Screening questionnaires were administered before the interview to collect information on participants’ symptoms of depression and anxiety, experience of school bullying, and demographic information.

##### **Depression**

We used the Chinese version of the 9-item Patient Health Questionnaire to assess participants’ symptoms of depression (PHQ-9) [25, 26]. Participants indicated the frequency of nine symptoms of depression in the past two weeks, using a 4-point Likert scale (0 = *Not at all* to 3 = *Nearly every day*). Items were summed for a total score, with higher scores indicating greater symptoms of depression. The Chinese PHQ-9 exhibited good internal reliability (*α* = 0.85) in the present study.

##### **Anxiety**

We adopted the Chinese version of the 7-item Generalized Anxiety Disorder Scale to measure participants’ symptoms of anxiety (GAD-7; [27, 28]). Participants reported the frequency of seven symptoms of anxiety in the past two weeks, using a 4-point Likert scale (0 = *Not at all* to 3 = *Nearly every day*). Items were summed for a total score, with higher total scores representing greater symptoms of anxiety. The Chinese GAD-7 exhibited good internal reliability (*α* = 0.86) in the present study.

#### Interview protocol

Individual interviews followed a semi-structured format, which is flexible to encourage rich data and is considered the exemplary data collection method for interpretative phenomenological analysis (IPA) [29]. A typical semi-structured interview is guided by an interview protocol with a set of questions to probe topics of interest and to facilitate an interactive dialogue by asking improvisatory follow-up questions based on participants’ narratives [30, 31]. The first and third authors co-developed the interview protocol through consensual discussion. The initial interview protocol was tested and revised based on pilot interviews conducted with two adolescents to ensure the flow of interview questions, participants’ understanding of the questions, and the ability of the interview protocol to elicit participants’ perspectives [32]. The protocol covers background information, the experience and impacts of bullying victimization, the process of seeking social support, and perceptions of supportive and unsupportive behaviors. Core questions, including sensitive topics related to experiences of bullying victimization, were positioned in the middle of the session to allow time for rapport-building and a “cool down” period at the end.

### Procedure

This study was part of a larger research project exploring school bullying among adolescents using a mixed-method design. All participants taking part in the research project were recruited from public middle schools in China by convenient sampling. The information sheet outlined the purpose of the study, what participation would involve, the voluntary nature of involvement, potential risks and benefits, confidentiality, and contact details for the research team. The definition and examples of school bullying were also included in the information sheet. When potential participants expressed interest, assent and consent forms were sent to them and their guardians via WeChat (a popular social media platform among the mainland Chinese population). After obtaining written assent and consent, potential participants were invited to fill in the online screening questionnaires.

Since IPA studies are committed to idiography and give equal appreciation to each participant’s account, they typically recruit small numbers of participants and concentrate on the richness and detailed account of each participant’s experiences [33]. To recruit a fairly homogeneous sample for IPA, we employed the following inclusion criteria: (a) Had a history of school bullying victimization in the current semester; (b) was 12–15 years old; (c) attended a day school; and (d) had been living with parents.

To protect vulnerable adolescents who may become distressed from discussing their bullying victimization experiences, adolescents who reported severe symptoms of depression (PHQ-9 ≥ 20) or anxiety (GAD-7 ≥ 15) were not invited to participate. In consideration of the sensitive nature of bullying, we implemented several measures to address instances of active bullying encountered during the study. If participants reported active bullying, we offered to inform school social workers about the situation. Furthermore, we provided a list of free, confidential school bullying and mental health hotlines, enabling participants to seek assistance while maintaining their anonymity. These steps were taken to prioritize the safety and well-being of participants throughout the research process.

Among the adolescents who expressed interest in this study, seventeen met the inclusion and exclusion criteria and were approached to participate. Seven did not provide consent or assent, resulting in a final sample of ten participants, which is a reasonable sample size for IPA research [34]. One-on-one interviews were conducted via audio call on WeChat and by the first author in Mandarin. Prior to the interviews, the interviewer received qualitative interview training on communicating with adolescents in an age-appropriate and culturally sensitive manner. Under the guidance of the third author, an experienced researcher in qualitative methods, the interviewer learned techniques for building rapport with participants, including expressing empathy, remaining non-reactive to disclosures, and being transparent and genuine.

At the beginning of each interview, the interviewer explained the meaning of confidentiality, how participants’ information would be used, and who would have access to the data. When adolescents appeared reluctant to respond to a specific interview question during the conversation, the interviewer refrained from probing further. After the interview, each participant received 100 RMB (USD 13.9) as a token of appreciation for taking part in the study. The amount was set at a level intended to acknowledge participation without exerting undue influence on either parental consent or adolescent assent. The interviews were audio-recorded and transcribed by the first author, with any identifying information removed to protect participants’ confidentiality. The average length of the interviews was 48.3 min (*SD* = 7.3). The study was approved by the authors’ institutional review board.

### Data analysis

The interviews were analyzed using IPA, which enables an in-depth examination of each participant’s lived experience of specific events (phenomenology) and the making sense of the experience by the participant (ideography) and the researcher (hermeneutics) [34]. The first and second authors followed Smith et al.’s [34] six-stage framework to analyse the data. First, the first author read the participant’s transcript and listened to the audio recording to help immerse in and actively engage with the data. She also reviewed notes taken during the interviews to recall the interview contexts, which helped highlight emotional tones and enhance immersion in the transcripts. Second, the first and second authors independently generate initial notes, including descriptive (e.g., content of statements), linguistic (e.g., repetition and pause), and conceptual (e.g., inferred values or beliefs) comments, to the transcript. For example, when a participant described feeling less burdened after confiding in friends about experiences of bullying victimization, this segment was annotated with the conceptual note “emotional support (from friends).” This exploratory process remained a phenomenological focus. To stay close to participants’ original expressions, descriptive notes were written in Chinese using participants’ own words. For instance, one note retained the phrasing “the teacher may not believe in me” (translated to English for reporting purposes). Third, the authors examined interrelationships and patterns between initial notes to develop emergent themes. This step signified a double hermeneutic in which the researchers tried to make sense of the participant who was trying to make sense of their lived experience [33]. Fourth, the emergent themes were organized into a table with line numbers corresponding to the transcript to allow easy cross-referencing. The authors then searched for connections across the themes, color-coded themes with conceptual connections, and cluster emergent themes to develop superordinate themes. As an illustration, several superordinate themes identified within one participant’s account included “psychological outcomes of school bullying,” “reason of not telling (bullying incidents to others),” and “perceptions of support received.” Fifth, the authors moved to the next participant and repeated the analytic steps for each of the ten participants. The authors approached each case ideographically to remain open to the unique sense-making within each participant’s account.

Sixth, after completing the tenth interview, the first author looked for patterns in the themes developed for each participant and organized higher-order themes for the whole group that captured both shared experiential features and divergencies. An instance of this was that, although participants described varying types (emotional, informational, instrumental) and valences (supportive and unsupportive) of support received, these accounts were integrated into a single higher-order theme capturing their evaluation of help-seeking outcomes. These higher-order themes formed the basis for determining whether the analysis had achieved sufficient richness for the aims of IPA. Although the concept of data saturation is an important consideration in qualitative research, the cyclical and iterative analytic process in IPA [34] indicates that the pursuit of complete saturation is not a suitable goal [35]. Nevertheless, the authors remained attentive to the recurrence of initial notes and emergent experiential patterns during the analysis of the later cases. The decision to conclude data collection after ten interviews was based not solely on such repetition, but on an assessment that the final higher-order themes had achieved sufficient depth, convergence, and meaningful divergence to address the research question in line with IPA’s analytic aims. The authors reached consensus that the final set of overarching higher-order themes demonstrated satisfying analytic sufficiency and that further recruitment was unnecessary.

#### Trustworthiness

Several methods were adopted to ensure the trustworthiness of this study. First, the authors discussed their expectations and opinions before analyzing the data, as past experiences and personal viewpoints may influence the research process and yield biased findings [36]. The authors believed that the process of decision-making could be complex and shaped by multiple factors. For example, the participants’ developmental characteristics might play a role in their decision-making process of seeking social support. Some adolescents may prefer to cope with school bullying on their own and turn to close friends as their primary or initial source for perceived social support. These considerations were framed as assumptions to be bracketed, serving to sensitized rather than predetermine, the subsequent analysis.

Second, the first and second authors independently developed notes and themes for the interview transcripts. Afterward, the two authors discussed regularly to settle discrepancies and reach consensus. When the authors discovered discrepancies in initial notes or emergent themes, they exchanged their rationales behind, revisited the relevant transcript excerpts together, and discussed alternative interpretations. Through this dialogue, they evaluated the fit of their interpretations and refined them until consensus was achieved. This process ensured that the developing themes and thematic framework reflected well-justified and collaboratively constructed analytic decisions rather than interpretations tied to a single perspective. The third author, who has rich experience conducting qualitative research, was consulted from time to time and provided feedback on the analyses. To guide the research process and facilitate reflexive thinking, the first author also engaged in regular discussion and consultation with the second and third authors throughout the analytic process. Third, all participants were invited to review the themes and corresponding quotes. Nine of them accepted the invitation and agreed with the themes and interpretation of the findings.

## Results

This study interviewed ten bullied adolescents to explore how they come to the decision of seeking or not seeking social support. Table 2 presents the four themes and nine subthemes emerged from the analysis.

**Table 2:** Qualitative results of bullied adolescents’ decision-making process of support-seeking

Themes and Subthemes	Frequency (%)
I. Prefer to use personal coping strategies	
A. Emotion-focused	8 (80%)
B. Problem-focused	9 (90%)
II. Determine whether the benefits of support-seeking outweigh the risks	
A. Benefits of seeking support	10 (100%)
B. Risks of seeking support	10 (100%)
III. Estimate if the threshold of support-seeking is reached	
A. Excessive psychological distress	6 (60%)
B. Severe or persistent bullying victimization	3 (30%)
C. Existence of external support	6 (60%)
IV. Evaluate the probable outcome of support seeking based on previous experiences	
A. Supportive behaviors and its effects	7 (70%)
B. Unsupportive behaviors and its effects	4 (40%)

### Theme 1: prefer to use personal coping strategies

Following the first few episodes of school bullying, all participants expressed a preference for using personal coping strategies. Their personal coping strategies can be classified as emotion-focused and problem-focused. In terms of emotion-focused strategies, eight participants reported to use attentional deployment (i.e., distraction techniques, wishful thinking) and four reported to use expressive regulation (i.e., suppression of negative emotions, venting). As for problem-focused strategies, nine participants noted to adopt passive coping (i.e., avoiding or escaping, being tolerant, and ignoring bullies) and four noted to employ active coping to stand up against bullying. All participants stated using multiple coping strategies simultaneously. For example, Jason (Boy, verbal bullying) said “I think I feel better every time I take a deep breath… I ignored what they [bullies] said about me.”

Participants selected personal coping strategies based on their bullying victimization experiences (i.e., the type, frequency, and impact of victimization). Their victimization experiences were, in turn, shaped by the personal coping strategies they employed. As such, there is a dynamic interaction between bullying victimization experience and personal coping strategies. For instance, Dawson (Boy, multiple forms of bullying) described how he reacted to name-calling:

At first, they [bullies] just occasionally called me names. I assumed they were joking so I did not take it seriously. I acted as though I did not hear it… And then, more and more people called me names… Later, they called me names whenever they saw me. I was irritated. When I am irritated, I do sports. I think sports could let go of my negative emotions.

### Theme 2: determine whether the benefits of support-seeking outweigh the risks

The preference to adopt personal coping strategies acted as an initial stage in participants’ response to bullying experiences. Besides personal coping, participants also weighed the benefits and risks of support-seeking to determine whether to reach out for social support. In terms of the benefits of support-seeking, six participants hoped that their parents or teachers can intervene directly to stop the bullies. Jason (Boy, verbal bullying) expressed, “I hoped they [my parents] can help me to solve this matter.” Five participants also valued the emotional comfort of social support. Maria (Girl, indirect and verbal bullying) said, “I felt very sad, so I wanted them [my close friends] to comfort me.” Moreover, three participants wished to learn how to cope with bullying from their parents. For example, Rose (Girl, multiple forms of bullying) noted that it would be a good idea to “listen to parents’ suggestions”.

On the other hand, participants were reluctant to ask for help because of the possible risks involved. All participants expressed concern about inappropriate responses, including a lack of understanding (e.g., invalidating feelings), ineffective teacher intervention (e.g., retaliation from bullies after teachers intervene), and inappropriate parental response (e.g., over-intervention). Another barrier to support-seeking pertains to adolescents’ self-presentation. Six participants believed that seeking support went against how they wanted others to see them, such as being independent, competent, and easy-going. For example, David (Boy, verbal bullying) said,

I am the monitor myself. I should provide assistance rather than creating troubles for the class teacher… Seeking support from the class teacher makes me seem incompetent… I have seldom told my family about my difficulties since I was a young child. Perhaps because I have grown up being tall, I am a boy, and now I am a big brother. I believe that I should handle most of the problems on my own, so I have rarely disclosed my struggles to my family.

Three participants also mentioned that a lack of trust stopped them from disclosing to their close friends and teachers. For instance, Rose (Girl, multiple forms of bullying) “did not completely trust” her close friends because she was concerned that her close friends might not keep secrets for her.

When considering the benefits and risks of seeking support, participants also took into account the severity of bullying and the effectiveness of their personal coping. For example, Jason (Boy, verbal bullying) expressed that when faced with occasional name-calling, he regarded such incidents as “not a big deal” and manageable on his own. At this stage, although he believed his parents could address the matter for him, he asserted that “reporting minor conflicts with peers to parents made him feel useless and incompetent”. However, as the frequency and severity of name-calling escalated, Jason stated that the circumstance was “beyond his capacity to handle” and that “his parents should be able to put an end to this [name-calling] more efficiently”.

Participants estimated the benefits and risks of seeking social support by drawing on their experiences from daily interactions. Three participants reported having regular and high-quality communication with their parents, which made them assume that their parents could support them when they were bullied. For example, Jack (Boy, verbal bullying) and his parents talked with each other nearly every day. His parents always offered solutions when he told them that something was bothering him. Hence, Jack believed that “they [his parents] could help to put an end to this matter [his bullying victimization experience]”. Furthermore, closeness in relationships also encourages support-seeking behaviors. For example, Rose (Girl, multiple forms of bullying) stated that she believed her grandma could offer emotional support because her grandma had been taking care of her ever since she was little and she felt close to her.

On the other hand, three participants noted that they were reluctant to ask for help due to a lack of trusting relationships. For example, Ryan (Boy, verbal and cyber bullying) “felt distant” from his teachers and refrained from talking his personal life with them. Additionally, two participants expressed reluctance to seek parental support because of the high value their parents placed on their academic achievement. For example, Rose (Girl, multiple forms of bullying) said, “I hesitated [to talk to my mother] because I felt that she might not understand my experiences… We should concentrate on our studies because we are middle school students. I worried that if I told my mother, she would blame me for paying attention to these things [school bullying] and say that I should have studied harder.” Past conflict also discouraged self-disclosure. For example, Anna (Girl, physical and verbal bullying) said,

I think they [my parents] would not understand… I once had a boyfriend. We had been together for three years. When my mother found out, she made us break up because she disliked him very much. We broke up at the end… They did not understand anything… I think they are bad to me. I have not spoken to them about myself since then.

### Theme 3: estimate if the threshold of support-seeking is reached

In weighing the benefits and risks of seeking support, adolescents simultaneously assessed whether their situation had reached a point at which external help was warranted. Before deciding to seek social support, participants needed to cross at least one of the three thresholds: Excessive psychological distress, severe or persistent bullying victimization, and existence of external support. Six participants reported that they reached out for social support when their psychological distress became overwhelming. For example, Nina (Girl, physical and verbal bullying) said,

Actually, it was incredibly difficult to carry it alone. I felt helpless. As soon as I returned to school, I started panicking. I had nightmares about them [the bullies]. I felt that I was at my breaking point. I was afraid that I would end up with some kind of mental illness, then my future will be totally ruined. So, I told my parents in hopes that they can help me. I also want to have someone to listen to me and comfort me.

Three participants stated that they looked for social support when they considered school bullying was out of control. For example, Jack (Boy, verbal bullying) was determined to seek support because he thought that, “I must fight back. If I do not fight back, more and more people will do that to [bully] me… When it gets out of control, it will be too late.”

In addition, five participants discussed the existence of external support during their process of support-seeking. Specifically, four participants noted to confide in their family members and friends when they were checked on. For example, Nina (Girl, physical and verbal bullying) said, “She [My mother] checked on me when she saw me crying in my room. I told her… My father came in my room later. Then I told both of them.” Two participants reported receiving interventions from bystander peers before deciding to seek external support. For instance, Anna (Girl, physical and verbal bullying) approached her class teacher because she was “persuaded by” and “taken to the teachers’ office by close friends”.

### Theme 4: evaluate the probable outcome of support-seeking based on previous experiences

Participants discussed their perceptions of the responses they received after seeking social support. Nine participants reported experiencing supportive behaviors and three mentioned unsupportive behaviors. Drawing on their perception of seeking support and its effects, participants evaluated the probable outcomes of continuing to seek social support in the future and adjusted their coping strategies accordingly. In this way, the consequences of support-seeking played a central role in reinforcing or recalibrating their initial preference for personal coping.

#### Supportive behaviors and their effects

The supportive behaviors reported by participants can be categorized as emotional, informational, and instrumental. In terms of emotional support, seven out of ten participants reported receiving verbal expressions of care and empathy. Specifically, they mentioned direct and indirect forms of verbal expressions. An example of direct verbal expressions includes, “We are here for you”. Participants reported feeling understood and reassured because they knew that “someone’s there for them”. For indirect communication, participants stated that hearing family and friends condemn (*n* = 2) and swear (*n* = 2) at the bullies validated their feelings and helped them feel empathized. In addition to verbal expressions, Zoey (Girl, verbal bullying) mentioned receiving nonverbal forms of care in the form of “tissues and hugs” after she disclosed to her family members.

Using distraction techniques to increase positive emotions were reported by three participants. Two participants mentioned that their parents took them out for fun to make them feel better. For example, Rose (Girl, multiple forms of bullying) said, “She [My mother] would come up with something to cheer me up and help me to forget about these things [bullying victimization experiences] … took me out to enjoy delicious food and have fun… I really liked it. It did divert my attention. My mood gradually lifted.” Another participant noted that her close friend used distraction to cheer her up too. Maria (Girl, indirect and verbal bullying) said, “[My close friend] did some weird dancing and sang with strange voices on the playground… She teased me and I laughed. Then I felt much better.”

As for informational support, all of the participants appreciated the suggestions they received because they felt cared for and taken seriously. Seven participants reported suggestions for dealing with bullying victimization. The most frequently reported suggestions were ignoring bullies (*n* = 4) and focusing on studies (*n* = 3). Four participants noted suggestions that help them to manage the impacts of bullying victimization. For example, after Zoey (Girl, verbal bullying) confided in her close friends about being upset over name-calling, her close friends gave her candy and told her that “having candy will make you feel better”. One participant also received suggestions on how to avoid potential physical harm. Jack (Boy, verbal bullying) told his parents that he was threatened by his classmates and they suggested to him, “call us immediately and we will come and pick you home, if someone threatens to bully you again.”

Regarding instrumental support, six participants reported receiving various forms of hands-on support in dealing with bullying, such as reporting bullying incidents to teachers (*n* = 4), criticizing the bullies (*n* = 3), and telling the bullies to stop (*n* = 2). Some of these actions helped the victims to improve the bullying situation (e.g., lessened the severity of school bullying, improved participants’ relationships with bullies, and fostered a positive classroom environment to stop bullying from happening), which made them feel reassured. For example, Jason (Boy, verbal bullying) said,

My classmates kept calling me names and I felt furious. I told my parents once I got home… My mother called my class teacher to report this matter right away. She also said that if they [my classmates] continue to call me that, she will call their parents… They no longer called me names… made me feel reassured. I knew that they [my parents] took it seriously and would help me to put an end to it. I feel that I can rely on them.

On the contrary, some of the interventions had less of an impact in stopping bullying, but the participants were nonetheless grateful for them. For example, Ryan (Boy, verbal and cyber bullying) said,

I told my friend that they [my classmates] have been calling me names in the online group. My friend is the class monitor… He requested all the classmates to stop calling me like that in the group… Although some of my classmates have continued to call me names, I think he has been helpful. He stood up for me in the group, which maintained my self-esteem.

#### Unsupportive behaviors and their effects

Three participants spoke about unsupportive behaviors from others after disclosing their bullying experience. There are two types of unsupportive behaviors: Emotional and informational. Regarding the emotional type of unsupportive behavior, two participants reported feeling as though their experiences and emotions were invalidated when asking for help. For example, when Nina (Girl, physical and verbal bullying) told her mother and teacher that she was upset and had trouble sleeping and focusing on her schoolwork, they responded by telling her that “you are just overthinking” and “it is not that bad”. She felt let down because she thought that neither of them knew how she felt. Moreover, one participant reported being treated impatiently after turning to friends for support. Dawson (Boy, multiple forms of bullying) said, “I told my two close friends [about my victimization experience] … they said, ‘Alas, look at you. You have been talking about this again and again!’… If I am bullied again, I still want to tell them, but I am afraid they will not understand.” When Dawson later went to his mother for support, his mother minimized his victimization experience by telling him, “You did not do well, why blame others?” He was let down by his mother’s response and attitude and stopped seeking support from her.

Two participants reported receiving unhelpful information in response to their seeking support. For example, Anna (Girl, physical and verbal bullying) said,

I was threatened by the senior student to wait outside the school gate in the afternoon. She would get her friends to corner me… My friends advised me to notify the teacher, so I reported to my class teacher. The class teacher said, ‘It is okay. You go out, they dare not to harm you. If they hurt you, you call the police.’ When I got out of school, they [the bullies] slapped me in the face… A few days later, they [the bullies] came to threaten me again… I did not tell the teacher this time. It is useless since he did not take it seriously. Besides, I am concerned that if the bullying escalates, those people will retaliate and continue to bully me.

Anna believed that her teacher did not take her words seriously, let alone put an end to her victimization experience, which made her feel disappointed and stop seeking support from him.

### Integration of themes: a dynamic decision-making process

Although presented as four distinct themes for analytic clarity, participants’ accounts reflected a recursive and evolving decision-making process unfolding across bullying victimization experiences. Rather than responding to a single event, participants continually reassessed their coping strategies as situations developed over time.

To illustrate, participants’ reliance on personal coping strategies (Theme 1) formed the immediate context within which further evaluations and actions occurred. Simultaneously, they engaged in two closely intertwined considerations: weighing the potential benefits and risks of seeking support (Theme 2) and judging whether the situation had reached a threshold that warranted external intervention (Theme 3). These two evaluative processes operated in parallel and informed one another. For example, perceptions of high relational risk could increase the threshold for seeking help, whereas severe or escalating incidents could lower the threshold despite anticipated risks. Importantly, these appraisals were context-dependent, shaped by incident severity, perceived availability of support, and prior interpersonal experiences.

Furthermore, participants evaluated the support they received and the outcomes of help-seeking (Theme 4). These outcome evaluations informed subsequent coping choices. In this way, the themes represent interdependent components of a cyclical decision-making system.

## Discussion

**Fig. 1:**
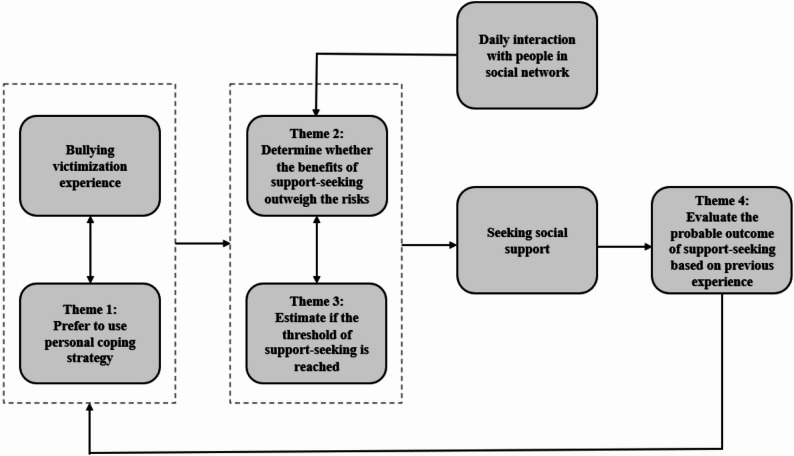
Bullied adolescents’ decision-making process of seeking social support

In this study, ten adolescents were interviewed about their experiences in deciding whether or not to seek social support after being bullied at school. IPA revealed four factors that underlie their support-seeking decisions: Prefer to use personal coping strategies, determine whether the benefits of support-seeking outweigh the risks, estimate if the threshold of support-seeking is reached, and evaluate the probable outcome of support-seeking based on previous experiences. Notably, these four factors and the interaction between them shaped bullied adolescents’ support-seeking decisions. Based on the findings, we developed a conceptual model to delineate bullied adolescents’ decision-making process of seeking social support (Fig. 1). We first discussed the findings factor by factor, followed by an integrated discussion of the decision-making process, with a focus on the interactions between the identified factors.

### Theme 1: prefer to use personal coping strategies

All participants stated that at the early stages of school bullying, they preferred to employ personal coping strategies, defined as strategies that rely on participants’ own resources, rather than seeking external support [37]. This likely reflects the developmental stage of adolescence, where the desire for autonomy and independence is prominent [5, 9, 38]. Driven by this strong desire to manage bullying independently, adolescents utilized both emotion-focused coping, which aims to regulate negative emotional reactions, and problem-focused coping, which seeks to alter or control the bullying situation. Furthermore, compared to adolescent girls, adolescent boys in our study felt more strongly that they should handle school bullying on their own. This may be related to the formation of male identity [39]. Boys tend to believe that being unable to cope with difficulties independently makes them appear vulnerable, thereby jeopardizing their socially constructed ideals of masculinity and male identity [4].

### Theme 2: determine whether the benefits of support-seeking outweigh the risks

All participants estimated whether the benefits of support-seeking outweighed the risks in their decision-making processes. On one hand, bullied adolescents turned to other people for emotion- and problem-focused support [1]. Specifically, participants valued having someone to comfort them, offer solutions, and help to stop their victimization circumstances. On the other hand, they reported various risks associated with support-seeking, including inappropriate responses, threat to one’s self-presentation, and fear of betrayal. Regarding inappropriate responses, participants worried that seeking social support may result in invalidation, ineffective teacher intervention, or inappropriate parental response. When bullied adolescents speak up, they hope for positive outcomes, especially when it has taken them some time and courage to do so. However, if their feelings are minimized and experiences downplayed, it will make them feel even worse [9]. Participants also regarded support-seeking as a threat to their self-presentation and peer acceptance. Particularly during adolescence, individuals place a high value on peer acceptance [40], and they employ various self-presentation tactics to align with their peer group and gain peer acceptance [41]. When it comes to school bullying, peer acceptance can also be a barrier to support-seeking because bullied adolescents who approach adults for support are sometimes stigmatized as “grass” or “mummy’s boy” [11 p82]. Consequently, bullied adolescents often hesitate to ask adults for help and would rather deal with it themselves to manage their self-presentation and to win acceptance from their peers.

To move forward, our study delved into how bullied adolescents form these assumptions and revealed that they assessed the benefits and risks of support-seeking based on their daily interactions with people in their social network. Positive daily interactions foster trust and closeness in the relationships that bullied adolescents have with people in their social networks, which in turn encourages them to seek support from these individuals [42]. Notably, only one participant in our sample sought social support from classmates. These observations align with Attachment Theory [43], which states that positive interactions foster secure attachments that promote support-seeking behaviors, while negative interactions generate insecure attachments that hinder adolescents from reaching out for help. When classmates are perceived as sources of stress in bullying episodes, these negative experiences deter bullied adolescents from seeking help from them.

A particularly novel finding in this study is that adolescents perceived parental emphasis on academic performance in daily interactions as an obstacle to support-seeking. Parental academic expectation has been extensively investigated in the field of education, but it is rarely discussed in relation to bullied adolescents’ support-seeking behavior. This finding is particularly relevant in societies deeply influenced by Confucian values, such as China, where education is viewed as the primary pathway to upward social mobility [44]. As such, there has been a longstanding cultural emphasis on academic achievement. Driven by these deep-seated beliefs, Chinese parents are highly invested in their children’s education and often demand excellence in academic performance [45]. Although higher parental expectations help adolescents perform better academically, they do not necessarily contribute to stronger parent-adolescent relationships or positive psychological outcomes in adolescents [46]. In addition, the present findings suggest that parental overemphasis on academic achievement may lead adolescents to believe that their parents are not interested in other aspects of their life, such as their social interactions at school. As such, even when the adolescents are bullied, they may be hesitant to seek support from parents if they think that they have not met their academic expectations. The cultural and educational context in China creates a complex dynamic where the desire to meet parental expectations may overshadow the need for emotional support even in the context of school bullying. This highlights the necessity of considering contextual factors when investigating adolescents’ support-seeking behaviors.

### Theme 3: estimate if the threshold of support-seeking is reached

Before seeking social support, bullied adolescents needed to cross at least one of three thresholds, or specific criteria or conditions that must be met, to seek social support. These include excessive psychological distress, severe or persistent bullying victimization, and existence of external support. Bullied adolescents typically initiate support-seeking when they perceive their emotional distress as beyond their ability to handle [12] or when they regard their situations as prolonged and severe [9]. Our findings reinforce this pattern and highlight that support-seeking is not solely internally motivated: Participants were more willing to tell others about their experience when someone checked on them. This indicates that support-seeking may be facilitated by external cues that signal genuine concern. Based on this finding, adolescents are more likely to get support from individuals (e.g., teachers, classmates, and parents) whom they are already familiar with, especially if these individuals take the initiative to extend support.

### Theme 4: evaluate the probable outcome of support-seeking based on previous experiences

After seeking social support, participants appraised the responses they encountered and reported both supportive and unsupportive behaviors. Three types of supportive behaviors were reported: Emotional (e.g., expressions of care and empathy), informational (e.g., suggestions to cope with school bullying), and instrumental (e.g., reporting bullying incidents to teachers). Participants’ narratives indicated that getting social support lessened the emotional distress and psychological burdens caused by bullying victimization. A noteworthy contribution of our study is that it demonstrates the existence of interrelationships between different types of perceived social support. For example, although our participants noted that not all bullying interventions (instrumental support) were successful in stopping bullying, they were still appreciative of them because the gesture of helping out made them feel that they were cared for and being taken seriously (emotional support). This observation suggests that instrumental support could carry emotional significance, a concept that has not received much attention in the literature [47].

Two types of unsupportive behaviors were reported by our participants. For the emotional type of unsupportive behavior, participants were disappointed when their experiences and feelings were invalidated and minimized. Participants felt let down and unsupported when they perceived the person that they turned to lacked empathy or understanding of what they were going through. Indeed, empathy and support are crucial in encouraging adolescents to seek support when they face difficulties [48]. Regarding the information type of unsupportive behavior, banalities were deemed as insincere and unhelpful. Participants who experienced either type of unsupportive behavior decided to stop seeking support from that particular person; some even stopped using social support as a coping strategy for dealing with school bullying.

### A holistic perspective towards bullied adolescents’ decision-making process

Based on the study findings, we propose a conceptual model to depict bullied adolescents’ decision-making process of seeking social support (Fig. 1). This model contributes to the school bullying literature by systematically organizing various factors influencing adolescents’ support-seeking behaviors, many of which have been extensively studied but not integrated into a cohesive framework. The model also complements existing support-seeking frameworks by providing a nuanced understanding of the decision-making process that bullied adolescents undergo when determining whether to seek social support. While traditional models, such as the Theory of Reasoned Action [15] and the Theory of Planned Behavior [16], outline general motivations behind support-seeking behaviors, they do not capture the specific challenges encountered by adolescents in bullying scenarios. Our findings reveal that factors such as the perceived effectiveness of personal coping strategies, self-presentation, and interactions with parents regarding academic performance play critical roles in shaping adolescents’ cognitive appraisals and subsequent decision-making processes. By incorporating these elements, our conceptual model captures the complex interplay between primary and secondary appraisals in school bullying.

Aligned with the primary and secondary appraisals central to the transactional theory of stress and coping [23], bullied adolescents’ cognitive appraisals determine their support-seeking decisions. Specifically, bullied adolescents’ primary appraisal involved assessing whether the situation is threatening (e.g., does name-calling pose a threat to me? ) and their secondary appraisal involved assessing whether the situation is manageable (e.g., are personal coping strategies effective? Is the situation out of control? Do the benefits outweigh the risks of seeking support from others? ).

Of most significance, the conceptual model extends the application of the transactional theory of stress and coping in understanding how bullied adolescents’ support-seeking decisions are shaped by the dynamic interactions between developmental factors (i.e., puberty) and contextual factors (i.e., school bullying). After the initial bullying incidents, adolescents appraised their victimization experiences (i.e., the characteristics and impacts of school bullying) and then selected personal coping strategies accordingly. Notably, there is a dynamic interaction between the appraisal of victimization experience and personal coping strategies. For example, bullied adolescents may view the first few episodes of name-calling as teasing between peers and the impacts as negligible. Thus, they choose to ignore the bullies. However, this passive coping strategy could contribute to more frequent and intense bullying victimization. Bullied adolescents may then perceive these new circumstances of school bullying as severe and the impacts as considerable, and subsequently may decide to stand up for themselves. As such, the appraisal of the bullying experience shapes the selection of the initial coping strategy, and the chosen strategy may change the bullying experience itself, which predicts changes in subsequent appraisal and coping.

In appraising their victimization experience and personal coping strategy, bullied adolescents also assessed the benefits and risks of seeking support and estimated whether they had reached the thresholds for doing so. For example, when adolescents perceive their victimization experiences as sporadic and minor, they tend to consider the risks of support-seeking as outweighing its benefits and do not seek social support. Conversely, when adolescents perceive their victimization experiences as prolonged and severe and their personal coping strategies as ineffective, they tend to prioritize the benefits over the risks of support-seeking and are more inclined to reach out for support.

After reaching out for social support, adolescents adjusted their coping strategies based on their appraisals of interactions with supporters and the perceived effects of the support. This iterative learning process underscores the role of interpersonal processes, which involve the dynamic interactions between individuals, including their feelings, thoughts, and behaviors [49]. Previous interpersonal interactions that adolescents experience with individuals in their social networks, both positive and negative, serve as meaningful learning experiences that shape their future decision-making regarding support-seeking. For example, if they think their interactions with supporters and the supporters’ responses will improve their victimization circumstances, they may appraise those responses as positive and supportive and may keep seeking support from these supporters as a coping strategy. However, if they consider seeking social support from a particular person as negative and ineffective, they will still reflect on these interactions as learning experiences. Consequently, they may turn to someone else for support or quit seeking support altogether.

### Practical implications

The findings of our study hold practical implications for school bullying interventions. First and foremost, adolescents’ cognitive appraisals shape how they perceive the identified factors of perceived social support and contribute to their decisions about seeking social support as a coping strategy for school bullying. Future interventions could focus more on promoting positive cognitive appraisals of support-seeking behaviors. For example, since bullied adolescents typically turn to others for emotion- and problem-focused support, highlighting potential benefits such as emotional validation and problem-solving may help shape their appraisals and reduce resistance to seeking support. At the same time, establishing a supportive and non-judgmental environment that normalizes support-seeking behaviors may lessen the associated stigma and possibly increase bullied adolescents’ willingness to seek social support. This could involve programs that provide guidance to teachers, students, parents, and social workers on how to foster open communication, take bullied adolescents’ concerns seriously, and attend to their needs. This is particularly crucial for adolescents who are at risk or already experiencing bullying, as creating a supportive environment can significantly enhance their willingness to seek social support.

Second, a more comprehensive understanding of bullied adolescents’ decision-making process helps us target not only at one factor but multiple factors simultaneously. For example, since adolescents gauge the potential benefits and risks of support-seeking based on their daily interactions with people in their social network, implementing programs that promote parent-adolescent relationships (e.g., Group Teen Triple P [50]) and teacher-student relationships (e.g., Establish Maintain-Restore [51]) would be beneficial. In addition, bullied adolescents in this study found it easier to disclose their experiences and ask for help when they were checked on by trusted others. It is therefore recommended that teachers, school staff, students, and parents receive training to enhance their awareness of school bullying, enabling them to spot school bullying and reach out to bullied adolescents in a timely manner. For adolescents who are at high risk of being bullied or currently facing bullying, timely outreach is essential, as it can help them overcome the threshold to seek help. One such example is the Olweus Bullying Prevention Program [52], which includes systematic training and hands-on practices to enhance people’s understanding of school bullying, heighten monitoring of bullying incidents, and provide effective responses to deal with school bullying. Furthermore, bullied adolescents reported encountering both supportive and unsupportive responses after seeking social support. Schools are advised to establish clearer guidelines and protocols for providing appropriate support in response to bullying. Guidelines and protocols should underscore the importance of emotional support, in addition to directly intervening in school bullying and providing effective and detailed suggestions. This could include, but is not limited to, expressing care and empathy towards victims and validating their feelings and experiences.

Equally important to encourage bullied adolescents’ support-seeking behavior is acknowledging their preference to handle school bullying on their own, as this is part of their developmental characteristics. Schools could help foster bullied adolescents’ adaptive coping strategies in response to school bullying. For example, the Coping with School Bullying program educates students to differentiate between effective and ineffective coping strategies and equips them with a repertoire of coping strategies suitable for different situations [53]. Programs like this can serve as a universal practice for all students, enhancing their capacity to employ both proactive and reactive coping methods in addressing school bullying. Furthermore, parents can inquire about their child’s social life at school in addition to their academic achievement, as overemphasizing academic success may discourage bullied adolescents from seeking support.

### Limitations and future directions

The present findings illustrate the interaction between various facilitating and hindering factors. Based on these findings, we develop a conceptual model to describe bullied adolescents’ decision-making process of seeking social support. However, the findings should be interpreted in light of the study’s limitations. Rooted in idiography, IPA research concentrates on in-depth exploration of the accounts of smaller samples. The small sample in this study may, therefore limit the generalizability of the findings. Specifically, all participants in our sample studied at urban schools and it is unknown the extent to which the identified factors and processes can be applied to bullied adolescents in rural areas. Compared to adolescents in urban areas, those in rural areas often experience more frequent school bullying while having limited support from home and school [54]. Furthermore, the sample was also relatively narrow in terms of bullying experiences, with many narratives centering on emotional and relational forms of victimization within close peer relationships. These contextual characteristics may have shaped the decision-making processes identified in the model, particularly around perceived risks, estimation of thresholds, reliance on peers, and judgments about when to involve adults. Different forms of bullying, varying developmental stages, and contexts with fewer safe sources of support may produce alternative pathways. Moving forward, future research may investigate the support-seeking experience among different samples to ascertain the applicability of the decision-making model.

Moreover, the “social network” examined in the current study primarily referred to offline ties within adolescents’ immediate school and family environments (i.e., parents, teachers, close friends, and classmates). As such, it does not represent the full range of social networks that contemporary adolescents engage with. In addition to these ties, adolescents may also seek support from extended networks, including people whom they interact with through online platforms. Support-seeking within online contexts may involve distinct considerations compared to offline interactions, including accessibility, confidentiality, and trustworthiness of available resources [55]. Given that the present study focused on offline relationships, the applicability of the proposed conceptual model to online support-seeking contexts warrants further investigation.

Quantitative studies may be undertaken with a larger sample of bullied adolescents to verify the decision-making model derived from our findings. Specifically, quantitative techniques, such as structural equation modeling, could be employed to examine whether and to what extent the identified facilitating and hindering factors predict adolescents’ decision-making. Another limitation of the study is that data were collected through a single interview with each participant. The richness and quality of information elicited from a single interview may be influenced by factors such as the participant’s emotional state, willingness to share, and rapport with the interviewer [56]. For example, bullied adolescents may conceal negative thoughts and opinions if they feel uncomfortable disclosing them to the interviewer. Future research could employ multi-session interviews to establish a stronger rapport with adolescent participants and gain deeper insights into their perceptions [56].

## Conclusion

Guided by the transactional theory of stress and coping, our study explores the interactions among factors influencing support-seeking and illustrates the decision-making process of bullied adolescents. The conceptual model developed in this study portrays a dynamic and recursive process, where bullied adolescents’ cognitive appraisals of multiple factors, along with the interaction among these factors, collectively shape their decisions regarding seeking social support. This understanding could help improve programs designed to encourage support-seeking behaviors in bullied adolescents.

## Supplementary Information

Supplementary Material 1.

Supplementary Material 2.

## Data Availability

The data that support the findings of this study are available on request from the corresponding author.
