# Supplementary material for: To seek or not to seek: decision-making in perceived social support among bullied adolescents
Source: BMC Psychol. 2026 Apr 1;14:692. doi: 10.1186/s40359-026-04474-w (PMC13169713; doi:10.1186/s40359-026-04474-w)
Supplement: Supplementary file 1 — Supplementary Material 1. [file 40359_2026_4474_MOESM1_ESM.docx]

**Appendix 1: Interview Protocol**

Thank you so much for agreeing to do this interview with me. Just to remind you that you choose to take part in a research project about social support of bullying victims. During the interview, I’d like to ask you a few questions which are mainly about how things have been going in your life, your understanding and experiences of dealing with difficult times. If you don’t want to answer a question or if it’s unclear then just let me know so we can skip it, or I can explain it. You are welcome to stop the interview at any time, without giving any reasons. The interview will be recorded and transcribed for data analysis, but any identifying information will be removed from the transcript to maintain confidentiality. We will use made-up names in our reports so that people don’t recognise you. Do you have any questions before we start the interview?

**Part 1: Background information**

1. Can you tell me about your school life?

  a. How is your relationship with your teachers?

  b. How is your relationship with your classmates?

  c. Do you have close friends outside school? Can you tell me a bit more about them?

  d. Do you have online friends? Can you tell me a bit more about them?

2. Can you tell me about your family?

  a. Who do you live with?

  b. How is your relationship with each person at home?

**Part 2: Experience of school bullying victimization**

In the questionnaire, you reported that you have experienced school bullying as a victim in the past 12 months (Questionnaire filled out before the interview).

1. Can you describe your experience with school bullying?

2. Is it still ongoing?

**Part 3: School bullying victimization and its effects**

1. How did you feel after the bullying victimization experience?

2. How does bullying affect your daily life?

3. How does bullying affect you psychologically?

4. Anything that you did to cope with these effects?

**Part 4: Perceptions of social support**

1. Who have you talked to about the bullying incidents?

* *Questions 2 to 6 will be asked of each person mentioned by the interviewee in Part 4 Question 1.*

2. How did you decide to talk to X?

3. How did X respond?

4. What do you think about X’s responses?

5. Have you told X how bullying affected you psychologically and your daily life?

6. If no, why did you not tell X about that?

** Questions 7 to 10 will only be asked if the participant told X about the psychological and/or daily life impacts of bullying.*

7. If yes, how did you decide to talk to X?

8. After learning these impacts, how did X respond?

9. Would you consider X’ responses to be helpful or unhelpful overall?

  a. How is X’s response helpful to you?

  b. (Refer to Part 3 Question 2) You said that you… because of the bullying incident. So

      how is X’s response related to improvement in your daily life? Can you give me

      some examples?

  c. (Refer to Part 3 Question 3) You mentioned that bullying made you feel… So how is

      X’s response related to improvement in how you feel? Can you give me some examples?

  d. Is there any part of X’s response that was not helpful? Can you give me some examples?

10. How do you think X could be more helpful?

* *Any of the interested sources of social support (i.e., teacher, parent/others living at home, classmate, close friend inside or outside school, online friend) that are not mentioned by the interviewee will be probed by Questions 11*.

11. When we talked about people who you looked for help, you didn’t mention Y (based

    on Part 1 Question 1 and Part 4 Question 1). What were the reasons that you decided not to talk

to Y?

   a. If you have told Y, how do you think Y would have responded?

We have come to the end of the interview. I would like to thank you again for doing this interview with me today. It’s been so helpful to speak to you and learn your perspectives. Do you have any additional comments? We are going to use these interviews to work out how best to help young people your age when their mental health is dampened by school bullying. Would you like to choose a pseudonym for when we write up our findings? This is a made-up name that we will use in our write-up to help ensure that other people don’t recognise you.
