# Supplementary material for: To seek or not to seek: decision-making in perceived social support among bullied adolescents
Source: BMC Psychol. 2026 Apr 1;14:692. doi: 10.1186/s40359-026-04474-w (PMC13169713; doi:10.1186/s40359-026-04474-w)
Supplement: Supplementary file 2 — Supplementary Material 2. [file 40359_2026_4474_MOESM2_ESM.docx]

**To Seek or Not to Seek: Decision-Making in**

**Perceived Social Support among Bullied Adolescents**

Felicia Huang^1^, Ruiqi Lu^2^, and Harold Chui^2^

^1^ The Jockey Club School of Public Health and Primary Care, The Chinese University of Hong Kong, Hong Kong SAR, China

^2^ Department of Educational Psychology, The Chinese University of Hong Kong,

Hong Kong SAR, China

**Author Note**


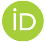
Felicia Huang <https://orcid.org/0000-0002-3209-9055>

[felicia.yanghua.huang@link.cuhk.edu.hk](mailto:felicia.yanghua.huang@link.cuhk.edu.hk)


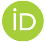
Ruiqi Lu <https://orcid.org/0009-0003-8756-6954>

[ruiqilu@link.cuhk.edu.hk](mailto:ruiqilu@link.cuhk.edu.hk)


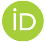
 Harold Chui <https://orcid.org/0000-0003-2066-8107>

[haroldchui@cuhk.edu.hk](mailto:haroldchui@cuhk.edu.hk)

Correspondence concerning this article should be addressed to Felicia Huang, The Jockey Club School of Public Health and Primary Care, The Chinese University of Hong Kong, Hong Kong SAR, China. Email: [felicia.yanghua.huang@link.cuhk.edu.hk](mailto:felicia.yanghua.huang@link.cuhk.edu.hk)
